# Machine-learning approach facilitates prediction of whitefly spatiotemporal dynamics in a plant canopy

**DOI:** 10.1093/jee/toaf035

**Published:** 2025-02-27

**Authors:** Denis O Kiobia, Canicius J Mwitta, Peter C Ngimbwa, Jason M Schmidt, Guoyu Lu, Glen C Rains

**Affiliations:** College of Engineering, University of Georgia, Tifton, GA, USA; College of Engineering, University of Georgia, Tifton, GA, USA; College of Engineering, University of Georgia, Tifton, GA, USA; Department of Entomology, University of Georgia, Tifton, GA, USA; College of Engineering, University of Georgia, Tifton, GA, USA; College of Engineering, University of Georgia, Tifton, GA, USA

**Keywords:** agriculture, deep learning, monitoring, pest management precision, vegetables

## Abstract

Plant-specific insect scouting and prediction are still challenging in most crop systems. In this article, a machine-learning algorithm is proposed to predict populations during whiteflies (*Bemisia tabaci*, Hemiptera; Gennadius Aleyrodidae) scouting and aid in determining the population distribution of adult whiteflies in cotton plant canopies. The study investigated the main location of adult whiteflies relative to plant nodes (stem points where leaves or branches emerge), population variation within and between canopies, whitefly density variability across fields, the impact of dense nodes on overall canopy populations, and the feasibility of using machine learning for prediction. Daily scouting was conducted on 64 non-pesticide cotton plants, focusing on all leaves of a node with the highest whitefly counts. A linear mixed-effect model assessed distribution over time, and machine-learning model selection identified a suitable forecasting model for the entire canopy whitefly population. Findings showed that the top 3 to 5 nodes are key habitats, with a single node potentially accounting for 44.4% of the full canopy whitefly population. The Bagging Ensemble Artificial Neural Network Regression model accurately predicted canopy populations (*R*² = 85.57), with consistency between actual and predicted counts (*P*-value > 0.05). Strategic sampling of the top nodes could estimate overall plant populations when taking a few samples or transects across a field. The suggested machine-learning model could be integrated into computing devices and automated sensors to predict real-time whitefly population density within the entire plant canopy during scouting operations.

## Introduction

Farmers continue to struggle with scouting small insect pests to make appropriate decisions for pesticide applications. An ongoing challenge is the silverleaf whitefly (*Bemisia tabaci*, Hemiptera; Gennadius 1889). Whiteflies impact agricultural areas that depend on cotton and other crops, such as vegetables, horticultural plants, or orchards, by damaging plants and transmitting over 300 plant viruses (Aslam et al. 2001, Babar et al. 2013, Afzal et al. 2023). Infestations of whiteflies in cotton fields may cause losses of up to 50% in case of not sprayed with pesticides (Babar et al. 2013, Cremonez et al. 2023). Furthermore, making appropriate management decisions requires sampling whitefly populations within plant canopies under field conditions, which is challenging (Aslam et al. 2001). Such challenges include (i) inspecting whiteflies beneath cotton leaves. Whiteflies mostly spend their time on the underside of leaves compared to other pests that congregate and/or feed on leaf tops, (ii) they are tiny in size of approx. 1mm (Barbedo 2014, Perring et al. 2018), requiring a hand lens for magnification; and (iii) they tend to be unevenly distributed within the plant canopy. The uneven distribution leads to little information on where to allocate or focus detection sensors during field scouting, (iv) the presence of windy and dynamic natural light reflections may interfere with whitefly scouting aids, such as the hand lens or camera vision systems, and (v) the limited field access as plant grow and becomes more difficult to observe (Naranjo et al. 1996). Scouting in several crop systems, if conducted, is currently achieved through manually counting whitefly nymphs and adults on specific nodes of a few plants within the field and extrapolating to make entire field-level decisions ([Bibr CIT0042][Bibr CIT0051], Kiobia et al. 2023).

Various manual sampling techniques are proposed for estimating whitefly populations in cotton fields. For instance, selecting 2 leaves of comparable age from the upper, middle, and lower canopy positions was used to determine the possible trap and barrier approaches to reduce whitefly density in cotton fields (Zhang et al. 2020). A similar method of counting weekly whiteflies per 3 leaves (top, middle, and bottom) was reported when evaluating cotton resistance traits against sucking insect pests (Zhang et al. 2020). In other studies, adult whiteflies were estimated mostly based on the top 7 fully extended leaves, the middle 7 leaves, and 6 bottom leaves of the plant canopy (Naranjo et al. 1996). On the other hand, a study suggests that whitefly populations can be estimated at one or multiple specific cotton nodes without scouting all node stems (eg using 3 to 7 nodes (Ohnesorge and Rapp 1986), fifth node (Naranjo et al. 1996), third node (Cremonez et al. 2023)). In addition, other studies reported the fifth leaf on the main stem from the top canopy to be densely populated with whiteflies’ eggs and nymphs rather than adult whiteflies (Chu et al. 2000). Differences in detecting eggs and nymph whiteflies are vital as nymphs extract substantial levels of sap from the plant and become the next mobile adult population to continue with the reproduction and infestation.

Unfortunately, most of the studies compare whitefly populations in 3 canopy sections (top, middle, and bottom) without going further to predict the entire plant canopy whitefly population. Few studies have indicated to predict the whitefly population mostly using the statistical and empirical models to develop the relationship between the whitefly population and the proportion of field infestation (Chiu et al. 2019). A binomial empirical model has been proposed to predict adult whitefly populations based on whiteflies found at the fifth node mainstem terminal (Naranjo et al. 1996). The model intends to detect the minimal thresholds of insects present in a leaf before such a leaf is declared infested. Their model was robust at 3 insect thresholds per leaf. However, those models may not be accurate for simulating adult whitefly populations when the fifth node leaves host more eggs or whitefly nymphs (Naranjo et al. 1997). The study showed that models are accurate when mean densities are less than 2 adults per leaf. At more intense densities, the model may overpredict the mean density. Another empirical linear regression model was developed to combine whiteflies counted in the top, middle, and bottom leaves (Aslam et al. 2001). Based on such empirical regression, the study recommends the number of whiteflies per middle leaf to be considered for pest management decisions. Random selection of one leaf from either top or middle or bottom sections per plant was also reported when forecasting and modeling sucking pests in the cotton agroecosystem (Hameed et al. 2014). The study utilized the Autoregressive Integrated Moving Average (ARIMA) model to estimate the relationship of rainfall, temperature, and relative humidity with the abundance of such cotton-sucking pests including whiteflies. However, while numerous studies have documented different methods to scout whitefly plant canopies, the use of machine learning to estimate the entire plant canopy adult whitefly population, and eventually the entire farm population using scouting information is still poorly understood. In addition, since most suggested models are either empirical or software-based, such models may have limited benefit when planning to design smart devices using sensors and embedded systems. Furthermore, studies explaining the distribution of the whitefly population within and between plants under field conditions are inadequate.

This study focused on analyzing the relationship between the full-canopy whitefly populations and the nodes with the highest densities of adult whiteflies, while also evaluating the accuracy of a machine-learning algorithm in predicting such a full-canopy population. The study examined the following: (i) adult whitefly main location relative to plant nodes (stem points where leaves or branches emerge), (ii) population variation within and between canopies, (iii) whitefly density variability across fields, (iv) the impact of dense nodes on overall canopy populations, and (v) the feasibility of using machine learning for prediction. Using such information may facilitate the design of automated sensors estimating adult whiteflies, which has the potential to promote more efficient and effective monitoring of the spatial distribution of whiteflies.

## Material and Methods

### Experimental Design and Plot Management

We conducted the study at the Horticulture Hill Research Farm, located at the University of Georgia, Tifton Campus, at an approximate latitude of 31.47°N and a longitude of 83.53°W from August through October of 2023. A daily count of adult whiteflies was conducted. To ensure a real farm environment, sufficient replication, and account for potential variability between plots, the experiment was conducted in 2 main fields (each measuring 300 ft in length and 50 ft in width) using a split-plot design. The 2 cotton fields were separated by an approximately 50ft wide peanut crop field, which served as a buffer between them. The peanut field was of a similar length (300ft) as the experimental cotton fields. One field was irrigated, and the other one was not irrigated. Each field comprised 8 plots, making a total of 16 experimental cotton plots. Each plot consisted of 14 rows, 30ft long with 36-inch row spacing. The 4 plots in each field were randomly selected for data collection, while the remaining 4 plots in each field were treated with pesticides to reduce the reproduction of whiteflies. The non-treated pesticide plots were separated from the treated ones by a buffer of 25ft for each field. The foliar application pesticide included the Assail 30SG (5 oz/acre) and Tundra EC (6.4 fl. oz/acre). From the 8 plots without pesticides, 8 plants were randomly selected from the 2 center rows of each plot for data collection, totaling (64) plants being scouted daily between July and October. To ensure continuous monitoring of the same randomly selected plant, 4 plants were flagged in the left center row and 4 plants were flagged in the right center row of each plot. Our fields were located within an experimental zone that included additional whitefly host plants for different studies. Such host plants included tomato, watermelon, and squash crops. The soybean crop field was located next to one of the fields approximately 30ft while the tomato, watermelon, and squash crops were about 350ft away from both fields.

### Estimation of Node and Entire Plant Whitefly Densities

**Fig. 1.**
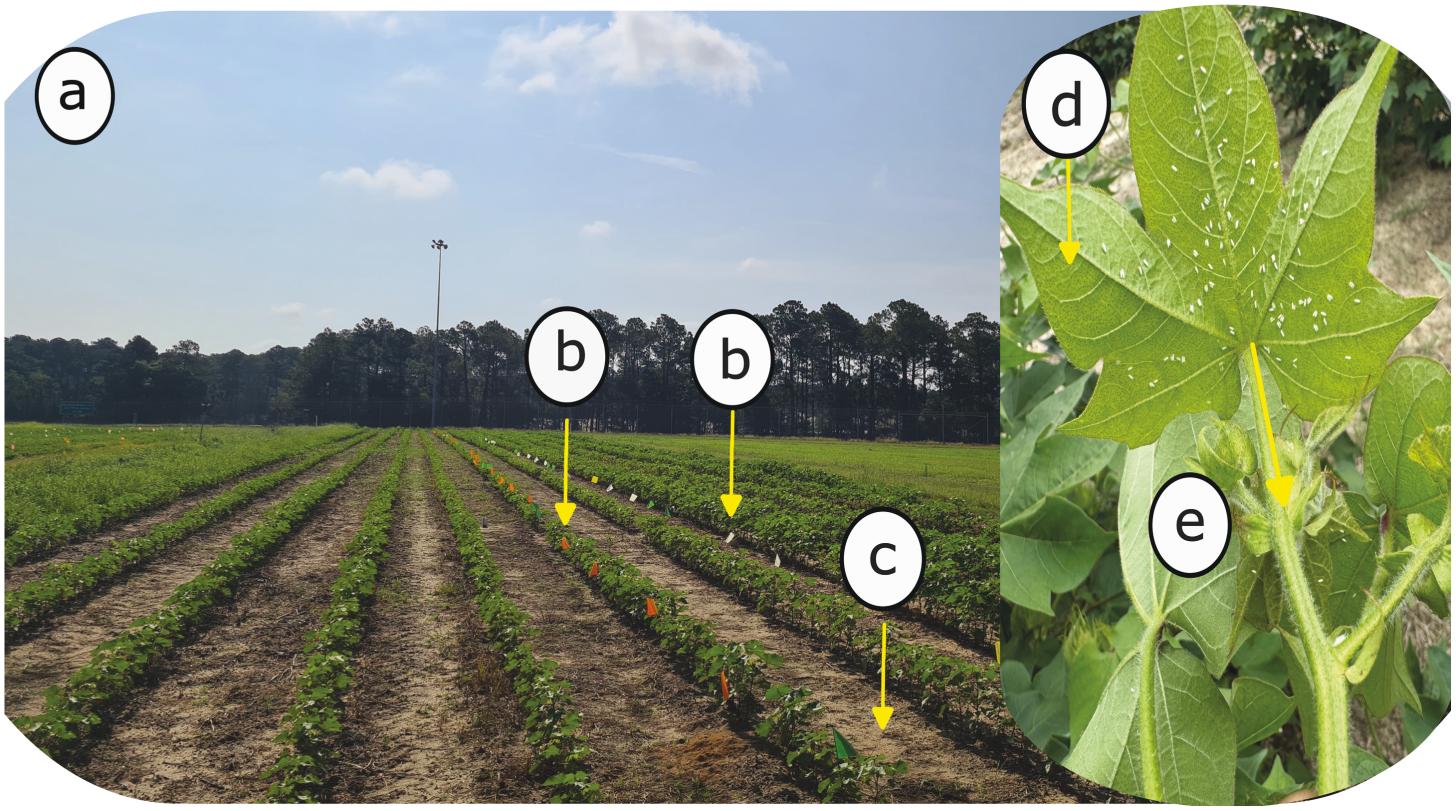
Determining the population of whiteflies in the entire canopy and most populated node. (a) Field and plot layouts, (b) experimental flagged plants, (c) monitored middle row for each plot, (d) Identification and decision of the leaves containing a higher whitefly population within the plant after counting the entire canopy whiteflies, and (e) Identification of plant node location hosting the leaves showing the highest number of whiteflies and then estimating total count of whiteflies in its leaves.

The abundance and total number of adult whiteflies in 2 cotton fields were estimated by using the visual counting method (Nadeem et al. 2022). The counting (Fig. 1) was conducted starting at 10:00 am to capture a higher number of whiteflies (Zhao et al. 2021). Additionally, it was decided to start at 10:00 to minimize the challenge of early morning plant wetness caused by dew on the leaves, which could make access to the plant canopy incovenient during data collection. In addition, scouting was not conducted on rainy days or weekends. During counting, the first step was to record the total canopy whitefly population. Our experiment involved counting the number of whiteflies on the entire plant, followed by counting the number of whiteflies on a specific node of the same plant. The counting started from the top to the bottom of the canopy (Ohnesorge and Rapp 1986). At this first step, all leaves on the entire canopy were inspected to obtain the whole canopy adult whitefly population (Rincon et al. 2016). After getting the total plant canopy population, the next step was to obtain the population count for the most populated node within the same plant canopy. To select the most populated node, we first identified the leaf with the highest number of whiteflies within the canopy, then located its corresponding node and counted the whiteflies on all leaves at that node (Fig. 1d,e). Therefore, the node population count included all leaves within the same node to get a more consistent and accurate assessment of the whitefly population at that specific node. When turning the leaf to observe its underside, care was taken to avoid disturbing the whiteflies being counted (N’cho et al. 2022). Moreover, light intensity (Lux) was also measured before taking data for each plot using the EXTECH light meter instrument (Model SDL 400, Wide range to 10,000Fc or 100kLux). The purpose of measuring the light intensity was to capture the conditions at the time of data collection for each plot, allowing us to evaluate the immediate effect of light on leaf visibility and its impact on whitefly detection when predicting the full-canopy whitefly population.

a) **Methodology for Analysis of Canopy Dynamic Distribution of Adult Whiteflies**

All analyses were conducted in R version 4.2.2 (R Core Team 2022). The total number of observations was 1664 for all plants over the season. After inspecting the data, whitefly counts were log-transformed to meet the constant variance and normality assumption. The transformation process involved the Box-Cox and Leven’s Test method to decide on the best transformation approach (Osborne 2010). We used linear mixed-effect models by employing the lmer() function from the lme4 package (Bates et al. 2014, West et al. 2022, Chen et al. 2024) to estimate the distribution of whiteflies within a plant and between plants before prediction. We treated the plant and corresponding node showing the highest number of whiteflies as random factors. The node factor was nested within the plant factor during the variability analysis (Boisgontier and Cheval 2016). When assessing the ranges of estimated variabilities, the mean square error within (MS_E_) and mean square error between (MS_A_) plants was calculated using a fixed-effect model fitted with the aov() function under the analysis of variance, ANOVA (Schmid et al. 2017). The estimation of variabilities in the whitefly population within and between plants, as well as the effect of highly populated nodes within the plant canopy, involved applying the following formulas, as indicated in Table 1.

**Table 1.** Estimation of variability components.

Estimation formula	Description of the estimated variability
$\delta^{2}=MS_{E}$	Variability within the plant. Estimated under random effects, using the lmer() function in R software
$\delta_{a}^{2}=\frac{MS_{A}-MS_{E}}{n}$	Variability between plants. Estimated under random effects, using the lmer() function in R software
$\theta =\frac{\delta_{a}^{2}}{\delta^{2}}$	Proportional variability between plants to variability within a plant
$\frac{SS_{E}}{\chi_{\alpha /2}^{2}\left(N-a\right)},\frac{SS_{E}}{\chi_{1-\alpha /2}^{2}\left(N-a\right)}\;$	A 95% CI for σ^2^
$\rho =\frac{\delta_{a}^{2}}{\delta_{a}^{2}+\delta^{2}}$	Intraclass correlation coefficient
$L=\frac{1}{n}\left(\frac{SS_{A}}{MS_{E}F_{\frac{\alpha }{2}}\left(a-1,\;\;\;N-1\;\;\right)}-1\right)\;$ $U=\frac{1}{n}\left(\frac{SS_{A}}{MS_{E}F_{1-\alpha /2}\left(a-1,\;\;\;N-1\;\;\right)}-1\right)\;$	A 95% CI for *θ*
$\frac{L}{1+L},\frac{U}{1+L}$	A 95% CI for the intraclass correlation coefficient *ρ*

MS_E_ = mean square error within plant, MS_A_ = mean square error between plant, n = number of observations per plant, *N* = total number of observations, a = number of plants under study, L = lower range, U = upper range.

(b) **Methodology for Prediction of Full Canopy Whiteflies Using Machine Learning**

### Description of the Tested Prediction Models:

The predictive performance of several predictive models requires tuning of one or more parameters (N’cho et al. 2022). The following models were compared in predicting the entire plant canopy whitefly population using predictor variables, including the location of the populated plant node, light illumination (lux), whitefly population count on leaves within a node, scouting time (morning or evening), and the daily condition (sunny or cloudy) at the time of scouting.

**Fig. 2.**
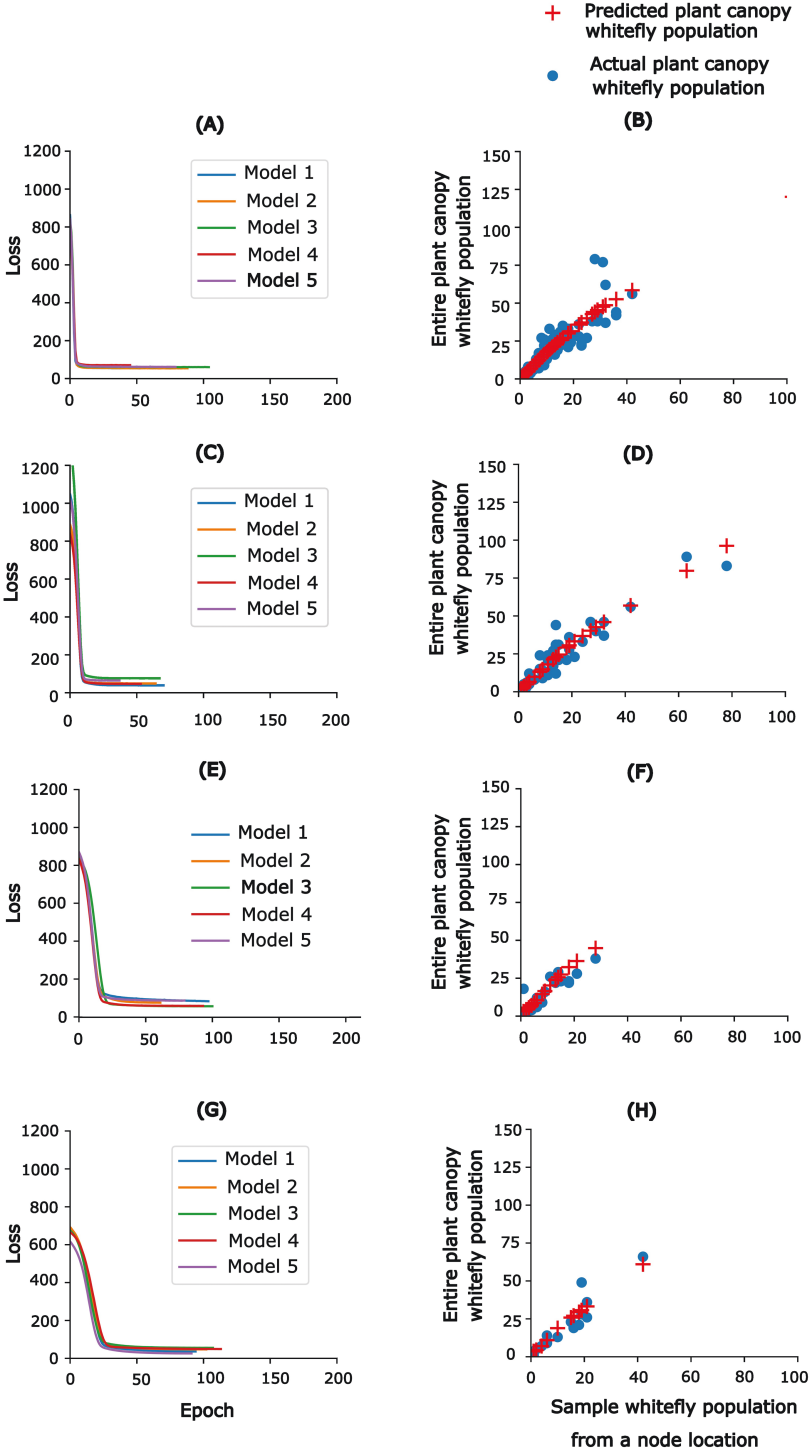
Behavior overview of the Bagging Ensemble ANN model when trained and tested in either pooled datasets or sub-datasets from whitefly host node locations. Each row of figures indicates the training and testing behavior of the model under a separate dataset. The left figure shows the training losses, and the right figure shows behavior during testing on separate datasets. The letters (A & B): Using all node-pooled datasets, (C & D): Using first top node sample dataset only, (E & F): Using second top node sample dataset only, and (G & H): Using third top node sample dataset only.

**Fig. 3.**
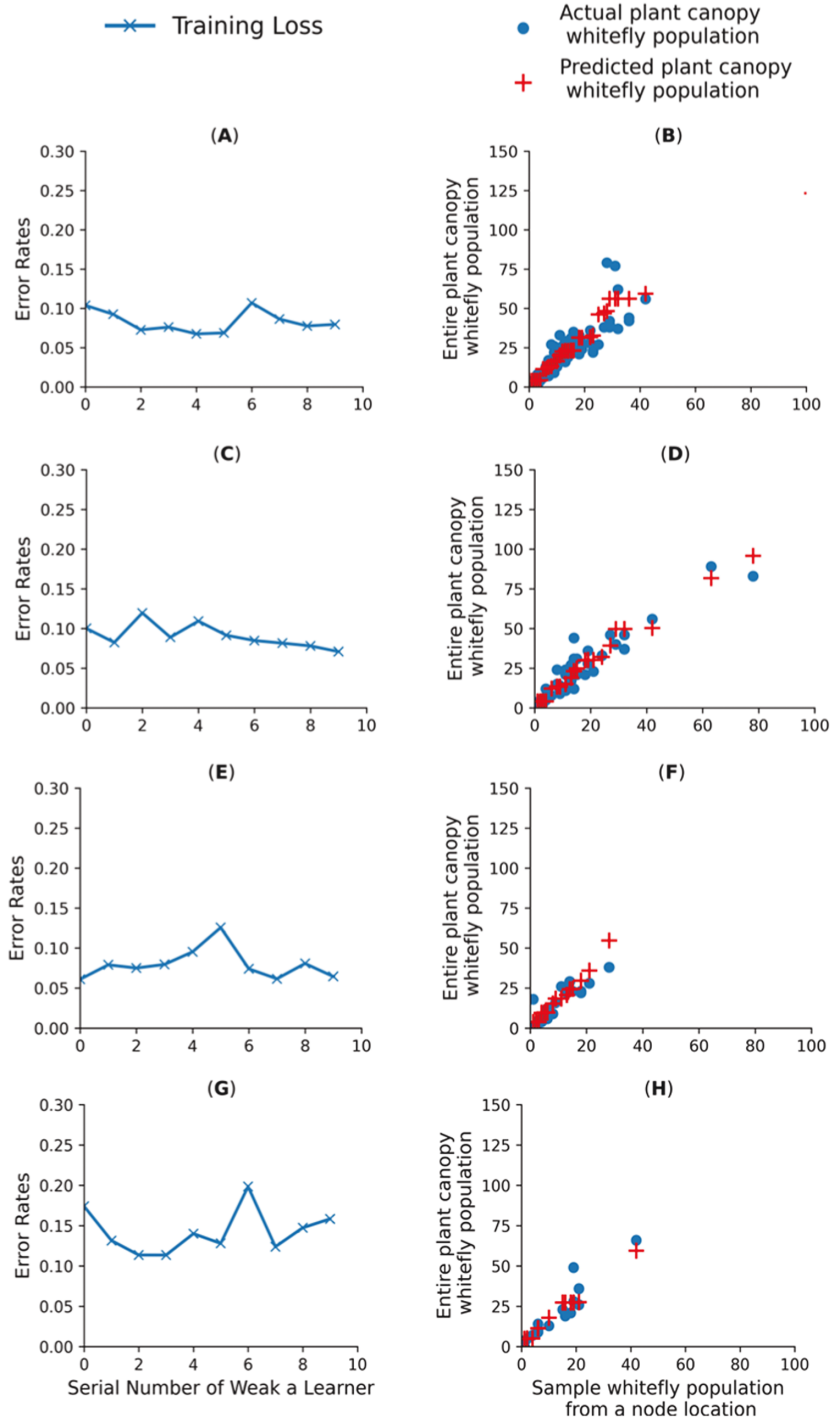
Behavior overview of the AdaBoost ensemble ANN model when trained and tested in either pooled datasets or sub-datasets from whitefly host node locations. Each row of figures indicates the training and testing behavior of the model under a separate dataset. The left figure shows the training losses, and the right figure shows behavior during testing on separate datasets. (A & B): Using all node-pooled datasets, (C & D): Using first top node sample dataset only, (E& F): Using second top node sample dataset only, and (G & H): Using third top node sample dataset only.

(a) Linear Regression: is a component of the general linear model (GLM) frequently used to predict one continuous variable from another continuous variable (Smith et al. 2013). A multiple linear regression can be produced by adding more variables to the basic linear regression. In this study, the ability of multiple regression in predicting the entire plant canopy whitefly population was evaluated.
(b) Random Forest: is an ensemble approach that uses Bootstrap Aggregation, often known as bagging, to combine several decision trees that are generated for regression tasks (Bratsas et al. 2019). First, the algorithm from the training dataset is used to determine the number of trees and bootstrap samples. Comprehensive regression trees are constructed for every sample. An optimal split is found from the most optimal subset of predictors rather than from all predictors at each node.
(c) Decision Tree Regression: is a collection of nodes and branches at each node (Pekel 2020). The technique solves nonlinear regression tasks by evaluating the value of features at each node and performing a binary split progressively. For each node, the winning feature is considered the discriminant. Splitting stops when the tree depth threshold or leaf node is reached (Nascimento et al. 2020). For this study, the optimal decision tree depth in reducing the error was 3 (3) at a cross-validation of 5 iterations. Using such tree depth helps reduce the overfitting of the model. In addition, out of the other inputs of the model, the node’s whitefly count was the only determinant at each depth and node.
(d) Artificial Neural Network is a network that effectively links several parameters with a broad variety of data points by imitating the structure of biological neural networks. Without complicated mathematical formulae, the models can relate several parameters (Xia et al. 2018). The model consists of neurons connected by adjustable fractional weights to minimize prediction error during training. By adding the value obtained from the sum of the input and output numbers divided by two, and taking the square root of the training point count, one may approximate the number of neurons (Garud et al. 2021). The overall number of neurons suggested for training, however, could also depend on the regularization technique and dataset complexity. The hidden layer, output layer, and input layer that are to be trained and evaluated need to be carefully selected to avoid the complexity of the model and reduce the use of maximum computational resources (Lu et al. 2011). According to research, a trained neural network is deemed appropriate for task prediction when it displays an error within a fair range (Chine et al. 2016). Any of the training functions, including Adam, RMSprop, stochastic gradient descent (SGD), and backpropagation, can be used by the model to determine the gradient of the loss function for the network parameters. The training function updates the parameters in a way that reduces losses as it maps inputs to outputs. Furthermore, depending on the nature of the dataset of a specific project, the output layer may also include any of the transfer functions, involving the Binary Step Function, Linear, Sigmoid, hyperbolic tangent function (tanh), rectified linear unit (ReLU), Leaky ReLU, Parametrized ReLU, Exponential Linear Unit, Swish, and SoftMax to optimize its performance (Sharma et al. 2017, Garud et al. 2021). Additionally, the model utilizes an iterative process (epochs) to optimize training, allowing the network to converge to an optimal solution, generalize unseen datasets, and monitor training progress. The artificial neural network (ANN) for this study was comprised of Adam’s training function, the ReLU transfer function, single hidden layers, and 500 epochs. In addition, the study evaluated the performance of neural network regression in the form of feed forward neural networks (FF-ANN), multiple perceptron layers (MPLs), and recurrent neural networks (RNNs). Based on the comparison findings, the MPLs were further evaluated when combined with either of the techniques including, the K-Fold validation Technique, Bagging ensemble, Regularization by L2 norm, and Boosting ensemble. Each model was tested under a node-pooled dataset and specific dataset from the main host nodes, including first, second, and third top nodes of the plant canopy. The purpose for testing each model on pooled dataset, and node specific dataset was to evaluate whether there is a need to recommend node specific machine-learning models, especially for the main whitefly host node locations. Since the artificial neural network ensembles were more promising based on the type of our dataset regardless of pooled dataset or node-based location dataset, our visualization and discussion on training and testing was confined to Bagging and Adaboost Ensemble techniques (Galar et al. 2011).
(e) Bagging Ensemble (Bootstrap Aggregating): creates many datasets from the original training dataset by using randomization and replacement sampling (Zhou et al. 2002). By employing replacement sampling and randomization strategies, the method makes sure that each dataset is the same size as the original. Some data points may not be selected, while the same data point may be chosen more than once when subsampling. With every sampled dataset, a distinct model of an ANN is trained, producing slightly varied outputs. The neural network architecture remains consistent across all models during training and testing. Each model has to be sufficiently accurate when contributing to the final prediction model (Alpaydin 2020). The technique aggregates the average predictions across the various individual models to produce the final prediction output. Combining predictions from different models reduces variation and increases stability. In this study, the original dataset was divided into 3 subsets training (70%), validation (20%), and testing (10%). Five distinct models of an ANN were used to train the model (Fig. 2a,c,e,g). Each model is trained using the Adam optimizer with mean squared error loss and 3 dense layers with ReLU activation functions. For testing purposes, each model was trained under early stopping conditions to minimize overfitting. Based on training with pooled datasets and training with node-specific datasets, the behavior of the Bagging ensemble model was observed during training and testing (Fig. 2).
(f) *AdaBoost:* is a machine-learning technique for binary classification learning problems (Shahraki et al. 2020). The structure of the method can be adjusted for continuous target variables rather than discrete class labels only. It creates a strong learner by combining predictions from several weak learners trained sequentially (Zhou et al. 2002). Training samples are given equal weight at first. Weak learners are trained one after the other, giving extra attention to the first incorrectly identified sample. The final prediction is based on a weighted mixture of inputs from weak learners. It works well on complex data, but its performance may vary with the amount of noise in the dataset. There is, however, some evidence that the performance of AdaBoost may vary depending on the amount of noise within the dataset of a given problem (Shahraki et al. 2020). Using 5 weak learners was found to be optimal in a study. Similarly, during training and testing, the behavior of the Adaboost ensemble model in terms of training loss and testing was observed simultaneously during training with a pooled dataset and training using a node-specific dataset (Fig. 3).

### Model training, Forecasting, Evaluation, and Selection process

The main scope was to create a data-driven model that predicts the presence of adult whiteflies throughout an entire plant canopy based on the count of the whitefly population in a plant canopy sub-sample. In this study, we used a single node as a predictor to obtain such a plant canopy subsample. The highest populated node includes counts from more than one leaf within one node.

Therefore, we utilized the scouting data to find an optimal model that can estimate the whitefly population at an individual plant level and eventually for the entire plant field in real-time during scouting operations. We evaluated linear regression and non-linear regression (machine learning) techniques and implemented the best model for the intended outcome. The process of achieving the best model through training and testing was conducted in 4 phases as shown in the flowchart (Supplementary Fig. S1).

#### Phase One

This phase evaluated the basic machine-learning models to predict the entire canopy whitefly population without considering the feature of importance. Such models included ANN Regression, Decision Trees (DT), Random Forests (RF), and Linear Regressions (LR). Each model was provided the input variables of the populated plant node, the light illumination intensity (lux) at the time of data collection, the number of whiteflies in the highest populated node, daily condition (sunny or cloudy), and the data collection time (morning or evening) at scouting time. The scaled data were split into 80% as a training dataset which amounted to 1332 rows and 20% as a testing dataset (~332 rows). The ability of each model to predict changes in whitefly density on an entire plant is compared based on low mean absolute error (MAE) and mean square error (MSE) predictions (Bratsas et al. 2019). The MAE and MSE are calculated as follows:


$$\begin{array}{l} MAE=\frac{1}{n}\overset{n}{\underset{i=1}{\sum }}|y_{i-}{\hat{y}}_{i}|\;\;and\;MSE=\frac{1}{n}\overset{n}{\underset{i=1}{\sum }}{\left(y_{i-}{\hat{y}}_{i}\right)}^{2} \end{array}$$

Where n is the number of instances in the dataset, y_i_ is the actual value of the target variable, for instance, i, and ŷ_i_ is the forecasted value of the target variable for instance i.

#### Phase Two

This phase included testing the performance of different ANN regression classes. Each model was trained and tested using all host node pooled data and specific host nodes. However, in this second phase, a feature selection process was conducted to avoid unnecessary inputs to the model. Choosing the right feature was not only critical to reducing the amount of input data required during scouting operations but also to saving computing resources. Based on outcomes from Random Forest, the whitefly counts in nodes holding the highest populated leaf was the only potential feature that could predict the entire plant whitefly density. Using the important feature selection, the study utilized the whitefly node population density as the only salient input feature in the rest of the model testing. Under phase two (2), we tested the performance of ANN regression model settings which included the Feed Forward Neural Network (FF-ANN), Multilayer Perceptron (MLP), and Recurrent Neural Network (RNNs). A similar approach was deployed to train and test each model’s performance. Aside from the MSE and MAE, the Coefficient of Determination and R-squared were added to measure the highest accuracy achieved with the plant section (node whitefly count) approach of predicting the total plant canopy whitefly count. The R-squared was calculated as follows:


$$\begin{array}{l} R^{2}=1-\frac{{\left(y_{i-}{\hat{y}}_{i}\right)}^{2}}{{\left(y_{i-}\bar{y}\right)}^{2}} \end{array}$$

Where ȳ is the average value of the target variables

#### Phase Three

Training and testing proceeded with the MLP Neural Network Regression model rather than FF-ANN and RNNs. However, the MLP relied only on multilayers without any improvement. Phase 3 of the study analyzed which of the K-Fold validation, Regularization L2 norm, Bagging ensemble, and Boosting ensemble techniques could improve the prediction of our traditional MLP. Similarly, each model was trained and tested using all host node pooled data and specific host nodes. The models were still examined based on the previous steps of MSE, MAE, and optimal R-square. Each model was still trained and tested using all host node pooled data and specific host nodes. The model built at this stage was considered accurate for real-time predictions for the entire whitefly population of the plant canopy, using whitefly counts from a highly populated plant node as a predictor variable.

#### Phase Four

The phase evaluated the effectiveness of the proposed model in predicting the entire canopy whitefly across various random sample dataset arrangements. In each evaluation, the predicted population count through a certain node location was compared to corresponding actual whiteflies that were counted manually during scouting. The initial evaluation involved predicting the entire canopy whitefly population based on randomly chosen node locations of a given plant. The model was tested using 10% samples from a specific node location, which included the first, second, and third nodes since were found as main location hosts in the scouted plant canopy during analysis. Subsequently, the second performance evaluation involved predicting the entire canopy whitefly population across randomly chosen samples for each scouting month. Similarly, ten percent (10%) of the samples were also randomly selected in August, September, and October. The third involved evaluating the ability of the model to predict the total whiteflies per plot. The total estimated whitefly count per season in each plot was compared to the corresponding manually observed entire canopy whitefly count per plot. Finally, the fourth performance evaluation involved the comparison of the daily entire plant canopy average whitefly population count against the corresponding predicted population. Such daily average comparison between the daily actual and predicted population was also evaluated based on the pooled dataset, and each dataset was from the first, second, and third top nodes.

## Results and Discussion

(a) **Results of the Analysis of Population Dynamics of Adult Whiteflies**

### Average Vertical Distribution of Whiteflies Within Plants

The average whitefly population in the entire plant canopy differed significantly from the average population in the plant nodes exhibiting a high whitefly population (*t* (1661) = 37.839, *P*-value < 0.0001) over the season. The total population density of whiteflies in the full canopy (Supplementary Fig. S2a) was greater than the whiteflies within the node of the highest number of adult whiteflies (Supplementary Fig. S2b). Subsequently, the grand mean whitefly population in the full plant canopy was higher (~22 per plant) than the average density of whiteflies found within a node indicating a high whitefly population (~12 per node). The incidence of seasonal plant whiteflies observed in the current study was significantly higher than the average seasonal whitefly count of 12 found in Pal et al. 2020. The average count of seasonal plant whiteflies reported in (Lu et al. 2011) was equivalent to the average number of node whiteflies (12) found in our study. Moreover, the seasonal whiteflies per plant observed in our study were comparable to the adult plant whitefly abundance value of 17.29 reported between 2014 and 2015(Didi et al. 2018, N’cho et al. 2022). The small deviations between the whitefly count per plant in this study and the previous study may be linked to differences in plant health, cotton variety, geographical location, and weather conditions, including rainfall, temperature, and relative humidity (N’cho et al. 2022). Such variability highlights the importance of plant-specific scouting within a season to account for the plant growth influencing factors.

The plants that indicated a higher average number of whiteflies also displayed greater standard deviations. The same variation was observed in the nodes with the highest percentage of whiteflies. Moreover, the number of total whiteflies within the node with the highest number of adult whiteflies reflected the number of whiteflies within the corresponding canopy. Comparing, plotsP1, P2, P3, and P4 were more infested than plots P5, P6, P7, and P8. Such differences may be associated with some parts of the fields experiencing different initial immigration rates of whiteflies (Boisgontier and Cheval 2016). Moreover, based on field observations, the densely populated plots had larger plant canopy in comparison to plots with lower populations. We speculate that such a large plant canopy had more fresh leaves, thereby fulfilling the requirements. The differences in natural enemy type and population among plots may also influence differences in whitefly abundance within plots (Abbas et al. 2023). Furthermore, some individual plants within and between plots had a higher whitefly density than others. The plants that indicated many whiteflies may also be considered as main hosts causing new plant infestation. Such observations have been reported in other crops, including cassava, where whitefly-infested plants may lead to new plants being infested daily during the season (Ferris et al. 2020). High wind speeds in the field may also contribute to the transfer of eggs and larvae from infested plant hosts to non-infested plants (Saleem et al. 2021).

### Distribution of Whiteflies in Plant Nodes

**Fig. 4.**
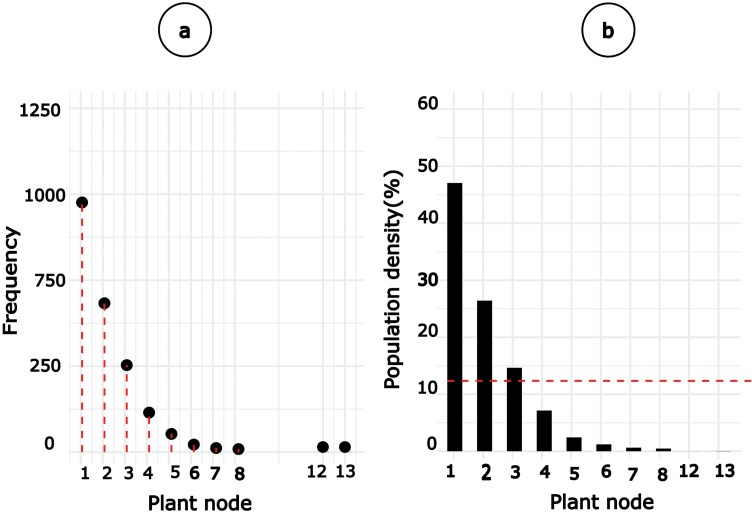
Seasonal distribution of adult whiteflies at various cotton nodes. Figure 4a shows the frequency of whitefly populations found at each node position, providing an overview of their occurrence. Figure 4b compares the percentage population of whiteflies at each node position. A red dotted line in Figure 4b highlights the average population of whiteflies at the most heavily infested node position. On the x-axis, “1” represents the top nodes, while “13” corresponds to the bottom nodes.

When scouting, many adult whiteflies were predominantly observed at the uppermost nodes of the plant canopy (Fig. 4a). Whiteflies were most common at the first node (about 750 times per 1664 observations), then at the second (2) and third (3) nodes. According to the population density at these nodes, the first node had the most whiteflies (over 40%), followed by the second and third nodes. Some plants possessed a reasonable number of whiteflies at the 4th to 13th node location, but such populations were below the average of 12 whiteflies per node location. Based on observations, the lower nodes may have more adult whiteflies, but the branches at these nodes are nearly the same height as the main stem, allowing the whiteflies to continue residing on the upper part of the plant canopy. Our results suggest that the cotton top section of the plant canopy, specifically nodes first, second, and third, or up to 5 were utilized during scouting as they indicate that they contain leaves hosting more adult whiteflies. The findings of this study closely align with previous research on cotton, which found a greater number of whiteflies at the top 7 nodes, compared to the middle or bottom nodes (Naranjo and Flint 1995). The populated nodes found in our study included the leaf on the fifth mainstem node, which is mostly reported to contain more whiteflies (Naranjo and Flint 1995, Naranjo et al. 1996). According to N’cho et al. 2022, the top 5 leaves may contain up to hundred (100) adult whiteflies before considering the population counts for the entire plant canopy. Furthermore, Naranjo et al., 1996 suggested that the plant could be considered infested if the fifth leaf on the mainstem contains more than 6 adult whiteflies. Such leaves in the top node of the plant canopy may be characterized as tender and contain adequate juice and nutrients as a feed for whiteflies (Kamble and Sathe 2015). Reaching top nodes manually or with scouting aids can be more practical while scouting than reaching bottom nodes. While some plants showed occasional observations of the fifth node (Fig. 4a), its population density was below the grand mean percentage (Fig. 4b). The lower density may be due to our study detecting adult whiteflies rather than eggs and nymphs. Our findings on the low density of the adult whitefly population at the fifth node location confirm the previous finding, except when scouting for nymph whiteflies (Chu et al. 2000, Zhao et al. 2021).

### Whiteflies Population Variabilities

The variability of the whitefly population inside a plant was greater than the variability among plants. Using the proportional measure (θ), we found that whitefly population density varied 9.7% less between cotton plants than inside them. This variability is predicted to vary between 6.1% and 16.4%. The intra-class correlation coefficient measure (p) verified the same results by calculating the ratio of whitefly density variability within plants to overall variability in the field. The variability across plants accounted for just 8.8% of the total population density variability across the entire field. Such variability among plants was found to range from 5.7% to 14.1%. The lesser variability between plants compared to the higher variability inside a single plant shows that when whiteflies spread, certain plants may be more seriously affected. On the other hand, it was found that a heavily affected node might contribute up to 44.4% of the overall plant whitefly population. The existence of severely whitefly-infested plants indicates the necessity for tailored scouting techniques to handle the unique issues provided by these plants.

(b) Results of Machine-Learning Model Prediction Performances

### Model Prediction Comparison

The main goal was to create a data-driven model that predicts the presence of adult whiteflies throughout an entire plant canopy based on the count of the whitefly population in a small section of the canopy. In this study, we used a single node that held highly populated leaves to predict the whitefly population within an entire plant canopy. Therefore, we used scouting data to find an optimal model that estimates the whitefly population at an individual plant level and eventually at the field level for real-time scouting operations. We evaluated linear regression and non-linear regression machine-learning algorithms and identified the most appropriate model for the intended outcome. Our findings showed that the following input features were most important to the model as follows: populated plant nodes (91.04%), light illumination intensity (lux) at the time of scouting (4.93%), whitefly count in the most populated node (2.59%), data collection time (morning or evening) (0.78%), and day condition (sunny or cloudy) during scouting (0.66%). Based on such observed percentages of features of importance, we identified the whitefly counts in the top 3 populated nodes as an important feature for developing the intended model. Furthermore, while the sunshine brightness condition was not the most significant feature, it closely followed the whitefly count in populated nodes. However, our study only factored in brightness during recording. A stronger correlation could be found when adding sunshine hours to scouting features, instead of just illumination brightness at recording time only (Kataria et al. 2019).

LR, RF, ANN, and Decision Tree are the main models used to predict continuous variables (Table 2). When provided with different scouting data inputs, the model may provide estimates of the entire canopy whitefly population density. However, the ANN regression model was the most accurate predictive model compared to LR and RF.

**Table 2.** Comparing the error rates of Artificial Neural Network, Random Forest, Linear Regression, and Decision Tree models for predicting whiteflies based on node population counts.

Model category	Mean squared error (MSE)	Mean absolute error (MAE)
Artificial Neural Network	**86.68**	**5.28**
Random Forest	94.33	5.70
Linear Regression	98.8	5.71
Decision Tree	126.35	6.69

The performance of ANN regression classes including Feed Forward Artificial Neural Networks (FF-ANN), Multilayer Perceptron (MLP), and Recurrent Neural Networks (RNNs) are expressed (Table 3, Group A). The table summarizes the performance of such predictive models only considering the whitefly node population density as the most significant input feature. Considering the same model selection criteria (MSE and MAE, R^2^), it was found that the MLP model outperformed the other FF-ANN and RNNs. The results indicated that MSE and MAE were consistently lower while accuracy (R^2^) was consistently higher whether tested with data from host node pooled data or a specific host node. However, MLP performance results still rely on multilayers without applying any improvement technique. Such results without data manipulation were not considered sufficient to explain the prediction of entire canopy whitefly density using single-node whitefly population density.

**Table 3.** Performance of types of Neural Network Regression algorithms on whole and piecewise data.

Group	Type of Neural Network Regression	Training and prediction based on the pooled nodes dataset			Training and prediction based on the first top node dataset			Training and prediction based on the second top node dataset			Training and prediction based on the third top node dataset		
		MSE	MAE	*R* ^2^(%)	MSE	MAE	*R* ^2^(%)	MSE	MAE	*R* ^2^(%)	MSE	MAE	*R* ^2^(%)
(A)	Multilayer Perceptron MLPs	43.27	4.78	85.39	62.72	4.56	83.93	96.72	5.22	79.21	59.71	5.70	79.64
	Feed Forward	56.96	5.42	80.56	62.35	4.20	84.02	112.29	5.61	75.86	56.98	5.40	80.57
	Recurrent Neural Networks (RNNs)	83.27	5.22	74.00	62.30	4.50	84.03	113.78	5.48	75.51	56.03	5.26	80.89
(B)	Bagging ensemble	**47.21**	**4.49**	**85.57**	**38.27**	**4.57**	**90.20**	**29.77**	**4.32**	**71.66**	**38.86**	**4.56**	**86.88**
	AdaBoost ensemble	54.28	5.09	83.41	40.00	4.85	89.50	40.57	5.14	61.37	47.68	4.86	83.90
	K-Fold validation	91.19	5.48	72.44	64.19	4.55	83.55	102.29	5.32	83.55	57.61	5.39	80.35
	Regularization by L2 norm	91.28	5.42	72.41	62.95	4.49	83.87	96.15	5.17	79.32	57.33	5.41	80.44

Group “A” represents the tested Neural Network Regression algorithm before the additional technique, and Group “B” represents the results after improving the MLPs with the additional technique. The highest score is highlighted in gray colour.

Our conventional MLP was improved by using K-Fold validation, Regularizing the L2 norm, Bagging ensemble, and AdaBoost ensemble techniques (Table 3, Group B). Compared to other types of regression ANN models, Bagging and Boosting models appear to be a good fit for our data. The Bagging ensemble technique yielded lower MSE and MAE and demonstrated superior R-squared compared to the Boosting model, irrespective of whether trained on all node pooled data or specific host node data. Similar results on higher performance of the Bagging Neural Network approach in comparison to Boosting Neural Networks have been reported. The reason for Boosting not performing better than the Bagging approach could be associated with the involvement of multilayers in the model design (Zhou et al. 2002). Bagging improved the entire canopy whitefly prediction performance by reducing the variance of the prediction error compared to a single-layer ANN (Khwaja et al. 2020). Considering the Bagging ensemble ANN model, it was feasible to consider the generalized Bagging model from pooled datasets rather than a node-specific model. The generalized Bagging ensemble model trained using all node-pooled data achieved the highest reasonable accuracy (*R*^2^ = 85%) compared to when trained using node-specific data. As a result, the generalized Bagging ensemble model showed potential for further testing under different data sample arrangements.

### Performance of Proposed Bagging Ensemble-ANN Model on Non-specific and Specific Node Random Samples

As part of the comprehensive evaluation of the generalized Bagging Ensemble-ANN model trained using pooled node data, the results in MSE, MAE, and R^2^ when predicting the entire canopy of whiteflies using random samples from each of the main 3 adult whitefly host nodes are observed (Supplementary Fig. S3). The model exhibited the highest accuracy when tested with samples from the first top node (*R*^2^ = 91%), followed by the second top node (*R*^2 =^ 88%), and the third top node (*R*^2^ = 74%). Similarly, the MSE and MAE were strong in the first top node dataset compared to errors recorded when prediction was conducted based on samples from the second and third nodes. The difference observed in the performance of the general model under specific node samples could be associated with a diversity of population counts within a specific node. In addition, the percentage contribution of the specific node in the training dataset to the generalized model could also be associated with the perceived performance differences. However, the accuracy observed in testing the proposed model under each node-specific sample was still sufficient to explain the entire canopy population. The role of host node whitefly counts in explaining the whitefly population was higher than the role of one leaf per plant revealed under a general multivariate regression model (Hameed et al. 2014). Most of the node samples exhibited fewer than forty whiteflies per node (Supplementary Fig. S3). Fortunately, the model was also successful in predicting such an adult whitefly population per sample. The observed accuracy for the node was comparable to the one reported by training the Radial Basic Function Network to predict whiteflies using environmental parameters, including temperature, humidity, rain, and wind speed (Saleem et al. 2021).

### Prediction of Proposed Model on Monthly Sample Data

For more insights into the performance of the recommended model trained using pooled node data, performance on a monthly sample dataset is demonstrated (Supplementary Fig. S4). Monthly sample data is also non-node specific. The percentage accuracy of the model in sample data for August, September, and October was 85%, 74%, and 81%, respectively. MSE and MAE were similarly lower in August, October, and September. The reason for the model expressing higher accuracy in samples obtained from August data may be linked to the peak abundance of adult whiteflies in August (Kataria et al. 2019).

### Model Prediction Performance per Plot

The coefficient of determination (*R*^2^) of the model suggested was greater than 80% for most field plots except plots 5 and 6 (Supplementary Fig.S5). Though the approaches were different, a similar coefficient of determination was achieved using yellow sticky tape to predict whiteflies in cotton fields (Pinto-Zevallos and Vänninen 2013). Also, the *R*^2^ value above 80% has been reported when adult whiteflies were predicted using a mathematical model under controlled greenhouse conditions (Parola-Contreras et al. 2022). Furthermore, the plots showed that the *R*^2^ values for plots 5 and 6 were 79% and 66%, respectively. The reduced coefficient of determination, especially in plot 6, may be attributed to increasing diversity in the whitefly population throughout the season across plot locations. Additionally, plot 6 had the lowest population density, probably leading to a small contribution to the model during its training. The analysis of the differences in the total number of seasonal whiteflies between the various plots revealed that the model overestimated for some plots (plots 5, 6, 7, and 8) and slightly underestimated for others (plots 1, 3, 4). However, the model also indicated nearly an identical estimate when considering plot number 2. Fortunately, the model still demonstrated a similar pattern by differentiating the densely populated plots (plots 1, 2, 3, and 4) from low-populated plots (plots 5, 6, 7, and 8).

Furthermore, the comparison in averages between the actual and the predicted whitefly population based on the two-sample t-test method in each plot is demonstrated (Supplementary Table S1). The results indicated that in all plots, the differences between the actual and predicted average populations in a plot were insignificant (*P*-value > 0.05). Hence, such results did not support the rejection of suggested estimates by the proposed Bagging Ensemble ANN.

### Prediction of Average Farm Whiteflies on Scouting Day

**Fig. 5.**
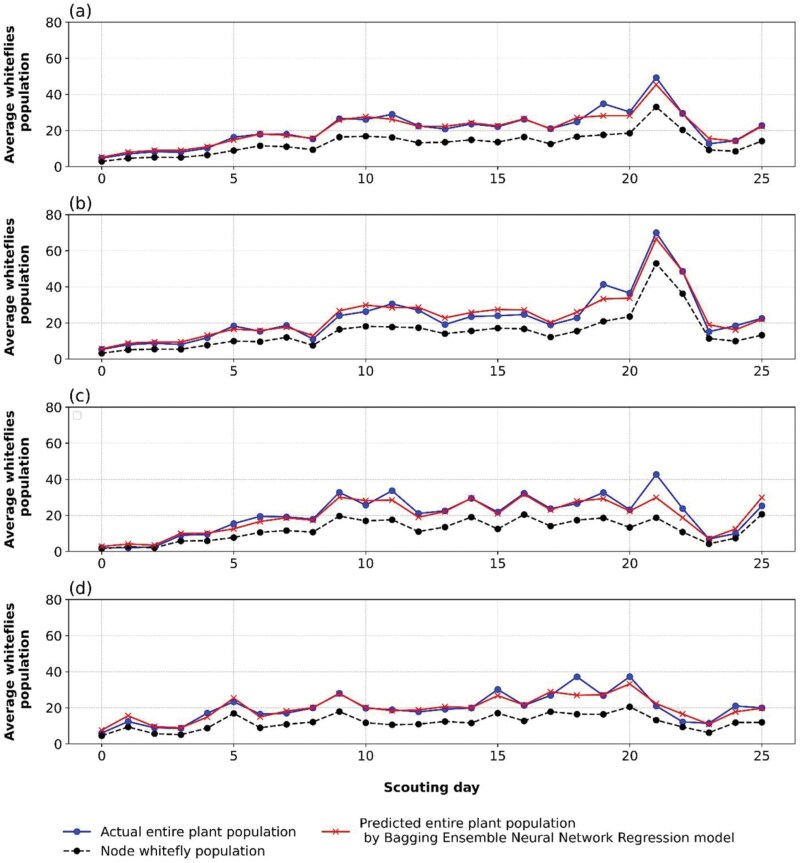
Prediction vs. Actual average farm adult whitefly population through densely populated plant nodes at each scouting day in a season. Fig. 5a: indicates general prediction through pooled nodes observed to contain leaves with a high number of whiteflies. Figures 5b, 5c, and 5d show predictions of whiteflies by counting them in leaves that appear at the first, second, and third top nodes, respectively.

The trend of the actual daily average whitefly population within the canopy (blue line), the daily average predicted by the proposed Bagging Ensemble -ANN model (red line), and the observed population at the host node (black line) are depicted (Fig. 5). The actual average population counts come from systematic field scouting. The whitefly count at the host node is an important input to the model, which helps estimate the total daily average whitefly population over the plant canopy. The average daily predicted whitefly population was nearly comparable to the average whitefly population recorded using the ARIMA model under meteorological conditions (Hameed et al. 2014).

Considering Figure 5a about the prediction performance based on the aggregated node whitefly population, the results reveal that over the first 5 d of scouting, the average number of whiteflies at any given node was similar to the daily average number of the entire canopy. The same observation applies to prediction using specific node populations at the first, second, and third whitefly host nodes as depicted in Fig. 5b–d, respectively. Such a pattern within 5 d may suggest that initially, the whitefly population was concentrated in a one-node section of the plant canopy. Such concentrated whiteflies on plants match prior findings, where the number of whiteflies in the aggregation is a decision factor in scouting based on recommended economic thresholds (ETL) of 5 or more adults or nymphs on a single leaf (Saleem et al. 2021). In scouting, the use of aggregated whiteflies to determine economic threshold levels (ETL) emphasizes the need for machine-learning models that are smoothly incorporated into embedded systems.

On the other hand, the daily average peak in the overall canopy whitefly population emerged predominantly on the 21st day of scouting (Fig. 5a). The same trend was observed even when considering the individual node populations at the first (Fig. 5b) and second (Fig. 5c) nodes. Based on these findings, the proposed model identifies such daily average whitefly population peaks in pooled and individual node populations. The reason for obtaining such a peak around the 21st day of scouting could not be quickly captured.

Our findings demonstrate interesting patterns in whitefly populations at various plant canopy nodes. Based on our research, we find that the dynamics we saw in the pooled-node population (Fig. 5a) were likewise mostly present in the first and second nodes but were less common in the third node. Comparatively, the first and second top nodes showed greater variations in average whitefly populations than the third node.

The average whitefly population for all pooled node populations decreased considerably on the 22nd and 23rd day of scouting, as Figure 5a illustrates. In the first top node (Fig. 5b), second (Fig. 5c), and third node (Fig. 5d), declining patterns are also observed for each node population. This drop is probably due to ecological variables, like the use of pesticides in nearby fields, which emphasizes the complex link between agricultural practices and the dynamics of pest populations.

In addition, whitefly populations increased once more throughout the entire plant canopy and each node after the 23rd scouting day. It turned out that this gain occurred at the same time as the plants were ready for harvest, which meant that our study was unable to fully document its magnitude. We postulate that a recovery of adult whiteflies occurred with the brief assistance of weather variations resulting in new, fresh leaves at the end of the season (Macfadyen et al. 2021). Understanding and anticipating these linkages is essential for longer-term, more specialized approaches to pest management.

In general, adult whiteflies are mostly found on the upper leaves of cotton plants. Moreover, whitefly population variability is higher within individual plant canopies than between plants. Furthermore, the whitefly population count in top nodes is a valuable indicator of the entire plant population count using machine-learning techniques. The Bagging Ensemble-ANN model is an effective scouting decision-making tool to predict the entire canopy whitefly population. The model exhibits a high coefficient of determination at low MSE and MAE. The Bagging Ensemble-ANN model has the potential to be integrated into an embedded computer system and convolutional neural networks to automate the estimate of canopy whitefly populations during pest-scouting operations. Such an approach may help produce more precise and accurate whitefly population data for improved pesticide management.

### Future Research Opportunities

The proposed Bagging Ensemble-ANN machine-learning model is feasible to predict the entire canopy adult whitefly population. However, since most management decisions are not only based on the adult whitefly population alone, we recommend also testing the suggested model on estimating the entire canopy whitefly population, either using the nymph population alone or in combination with adult whiteflies. In addition, the performance of the proposed model was evaluated by estimating the entire canopy whitefly population, using whitefly counts from multiple leaves at one location. Still, there is a need to estimate the entire canopy whitefly population based on other sampling techniques. Lastly, even though the model can estimate the cotton canopy whitefly density, there is still a need to evaluate its performance by including different cotton varieties, and other host crops, as well as weather parameter factors such as temperature, and sunshine hours as inputs to the model.

## Supplementary material

Supplementary material is available at *Journal of Economic Entomology* online.

toaf035_suppl_Supplementary_Material
