# Supplementary material for: Machine-learning approach facilitates prediction of whitefly spatiotemporal dynamics in a plant canopy
Source: J Econ Entomol. 2025 Feb 27;118(2):732–45. doi: 10.1093/jee/toaf035 (PMC12034313; doi:10.1093/jee/toaf035)
Supplement: toaf035_suppl_Supplementary_Material [file toaf035_suppl_supplementary_material.zip › Supplementary information.docx]

**Supplementary information**

**Supplementary Table 1**

*Supplementary Table S1: differences between actual and predicted whitefly populations in each plot*

**Supplementary Figures (1, 2, 3, 4, 5)**

***Supplementary Fig. S1****: Flowchart indicating the process for selecting and implementing an optimal model to predict the entire adult whitefly canopy based on scouting information.*

***Supplementary Fig. S2****: The average seasonal distribution of adult whiteflies within plants in each plot. Supplementary Fig. S2a illustrates the profile of average whiteflies found throughout the entire canopy. Supplementary Fig. S2b shows the profile of average whiteflies in plant nodes of many whiteflies. Each bar within a plot represents the monitored plant. The error bars show the standard deviation during the season. The blue dashed line represents the grand mean of the population density of whiteflies.*

***Supplementary Fig. S3****: The prediction performance of the proposed general Bagging Ensemble ANN model on both non-specific and specific main host whitefly node samples. a: Prediction is using the whitefly count sample from the first top node, b: using the whitefly count sample from the second top node, and c: using the whitefly count sample from the third top node.*

***Supplementary Fig. S4****: Performance of Bagging Ensemble ANN model under monthly non-specific node samples.*

***Supplementary Fig. S5****: Comparison between manual and predicted plant canopy whiteflies over the season using Begging ensemble artificial neural networks. R^2^ indicates how accurate a single node whitefly count is as a predictor variable for the entire plant canopy whitefly count. The prediction was conducted based on any main adult whitefly node host that was found in the given plot.*
