# Supplementary material for: Machine-learning approach facilitates prediction of whitefly spatiotemporal dynamics in a plant canopy
Source: J Econ Entomol. 2025 Feb 27;118(2):732–45. doi: 10.1093/jee/toaf035 (PMC12034313; doi:10.1093/jee/toaf035)
Supplement: toaf035_suppl_Supplementary_Material [file toaf035_suppl_supplementary_material.zip › Supplementary Table S1.docx]

*Supplementary Table S1: differences between actual and predicted whitefly populations in each plot*

| **Plot**  **number** | **Actual**  **average** | **Predicted**  **average** | **t-value** | **Degree of freedom** | **p-value** |
| --- | --- | --- | --- | --- | --- |
|  |  |  |  |  |  |
| 1 | 29.62 | 27.50 | 0.8662 | 414 | 0.3869* |
| 2 | 22.67 | 23.07 | -0.2226 | 414 | 0.824* |
| 3 | 24.75 | 24.01 | 0.3952 | 414 | 0.6929* |
| 4 | 28.41 | 26.47 | 0.8857 | 414 | 0.3763* |
| 5 | 16.49 | 17.44 | -0.7145 | 414 | 0.4753* |
| 6 | 13.96 | 15.37 | -1.1807 | 414 | 0.2384* |
| 7 | 16.48 | 17.97 | -1.0772 | 414 | 0.282* |
| 8 | 14.31 | 16.032 | -1.5185 | 414 | 0.1297* |

***** Indicates that the true difference between the actual and predicted average per plot is not equal to zero. The negative value on the t-value shows where the plots in which the model overpredicted the average whitefly population.
