# Supplementary figures and images for: Machine-learning approach facilitates prediction of whitefly spatiotemporal dynamics in a plant canopy
Source: J Econ Entomol. 2025 Feb 27;118(2):732–45. doi: 10.1093/jee/toaf035 (PMC12034313; doi:10.1093/jee/toaf035)

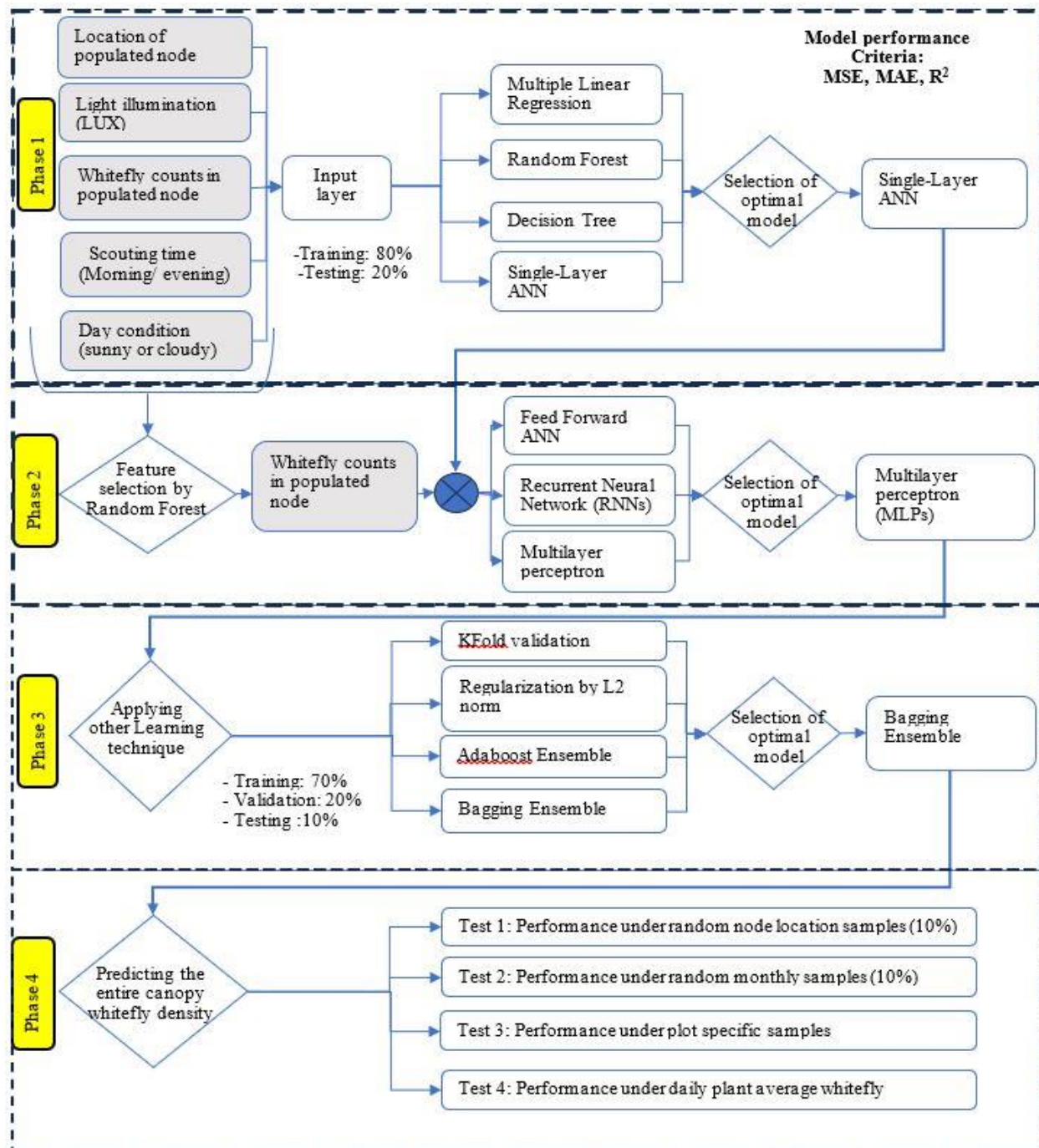

Supplement: toaf035_suppl_Supplementary_Material [file toaf035_suppl_supplementary_material.zip › Supplementary Fig S1.pdf]

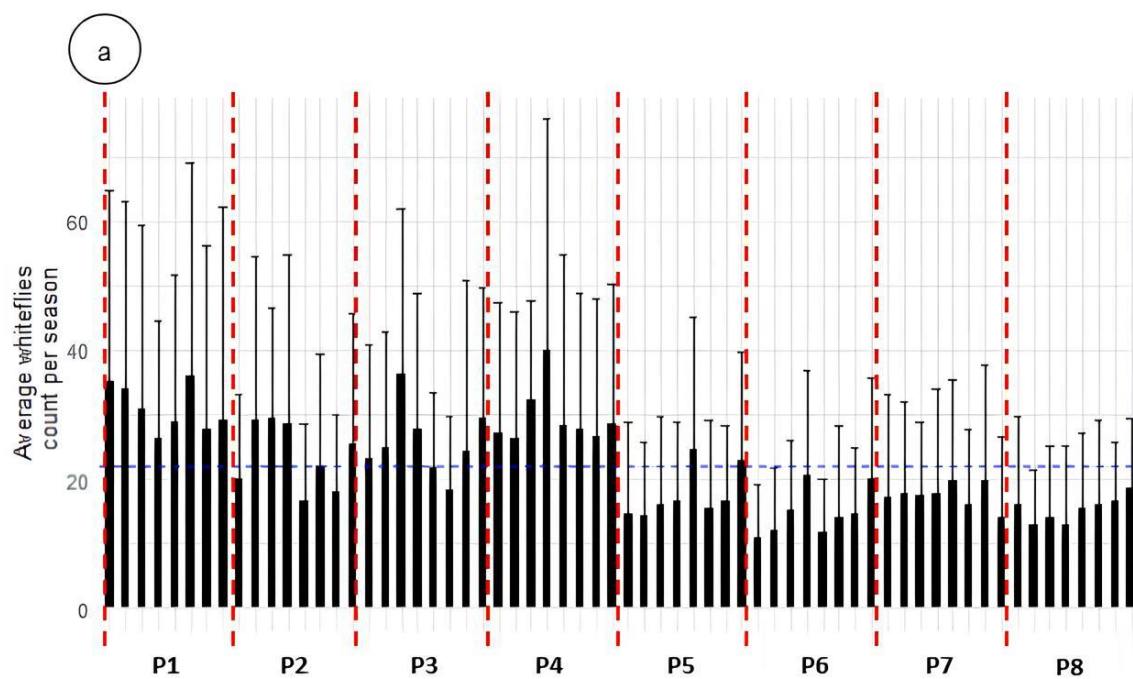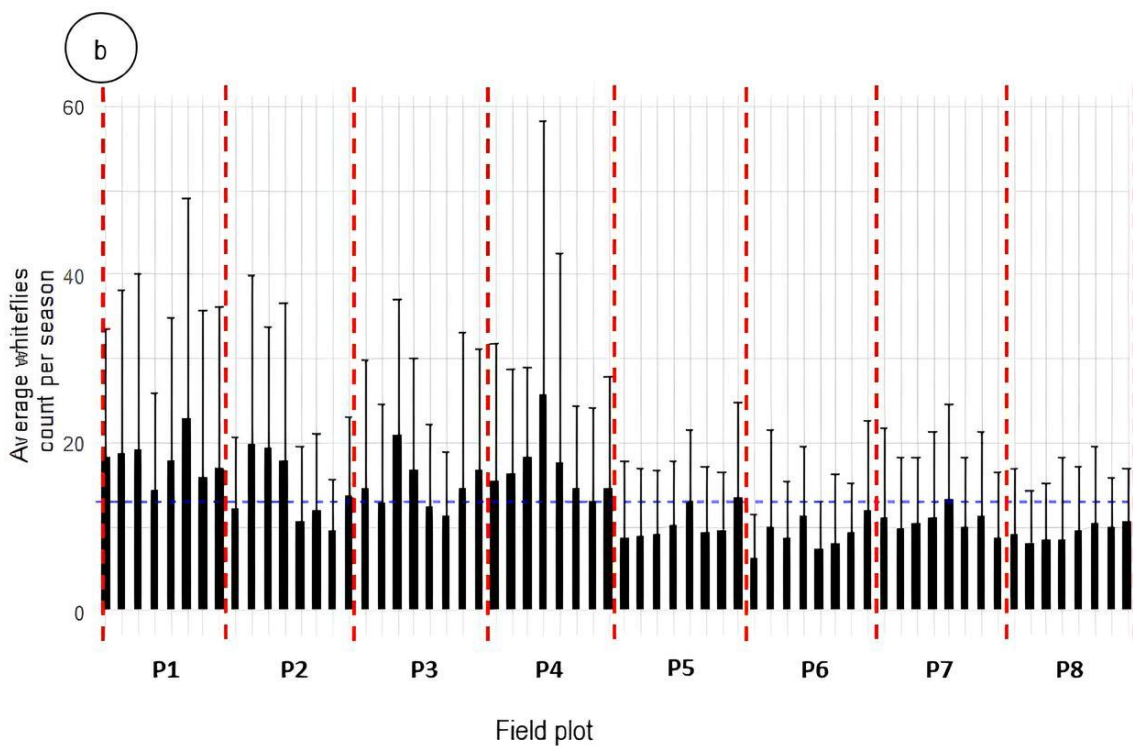

Supplement: toaf035_suppl_Supplementary_Material [file toaf035_suppl_supplementary_material.zip › Supplementary Fig S2.pdf]

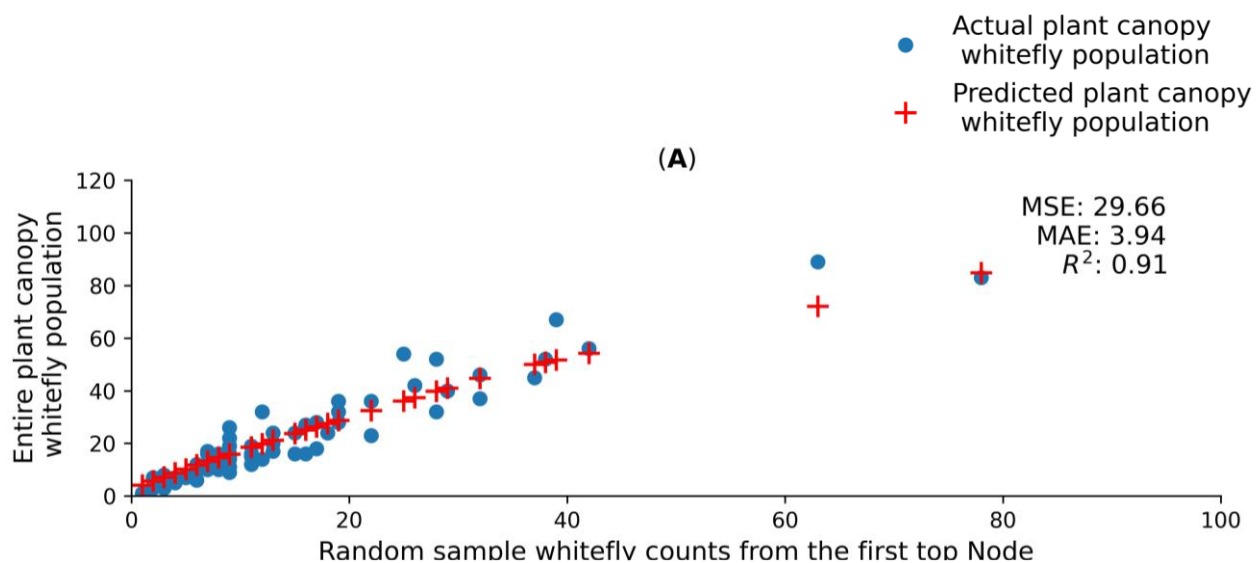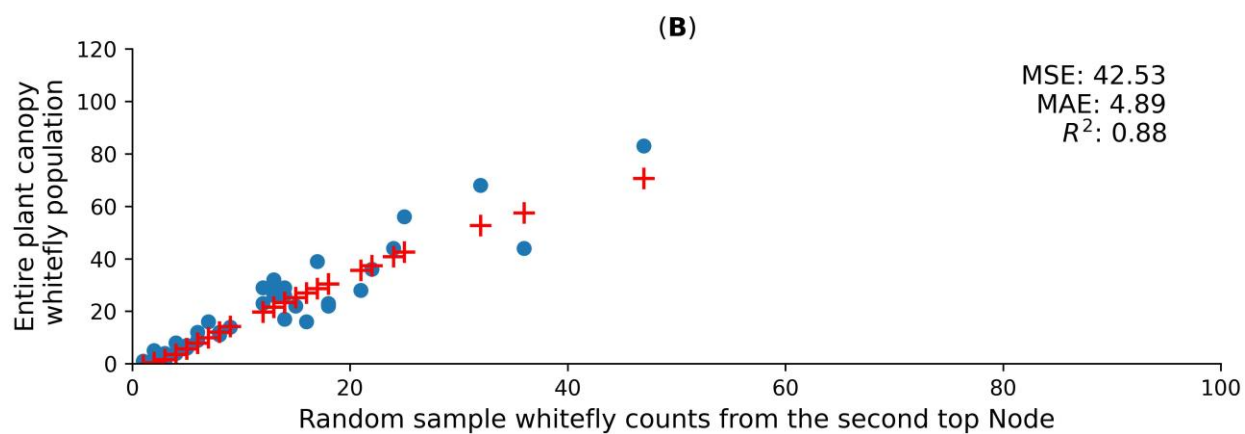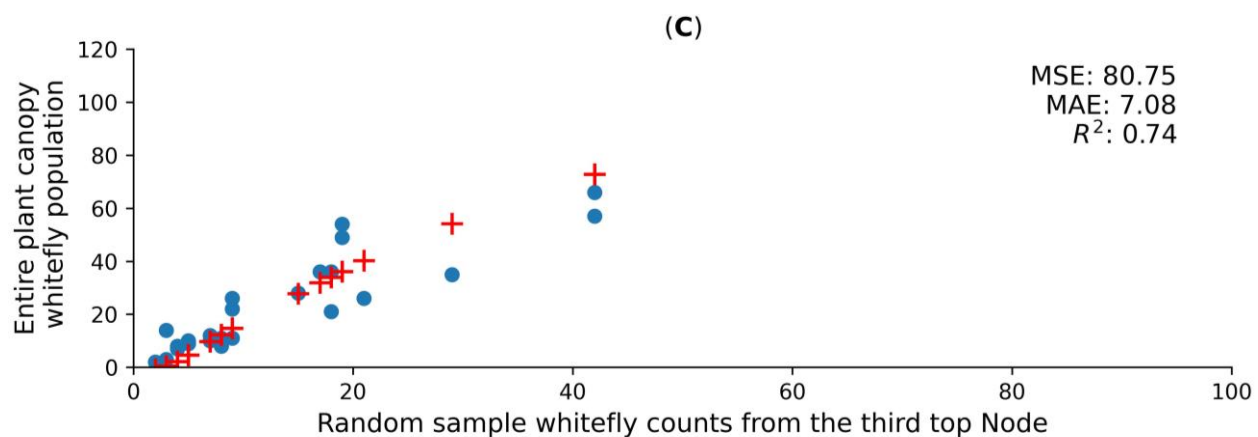

Supplement: toaf035_suppl_Supplementary_Material [file toaf035_suppl_supplementary_material.zip › Supplementary Fig S3.pdf]

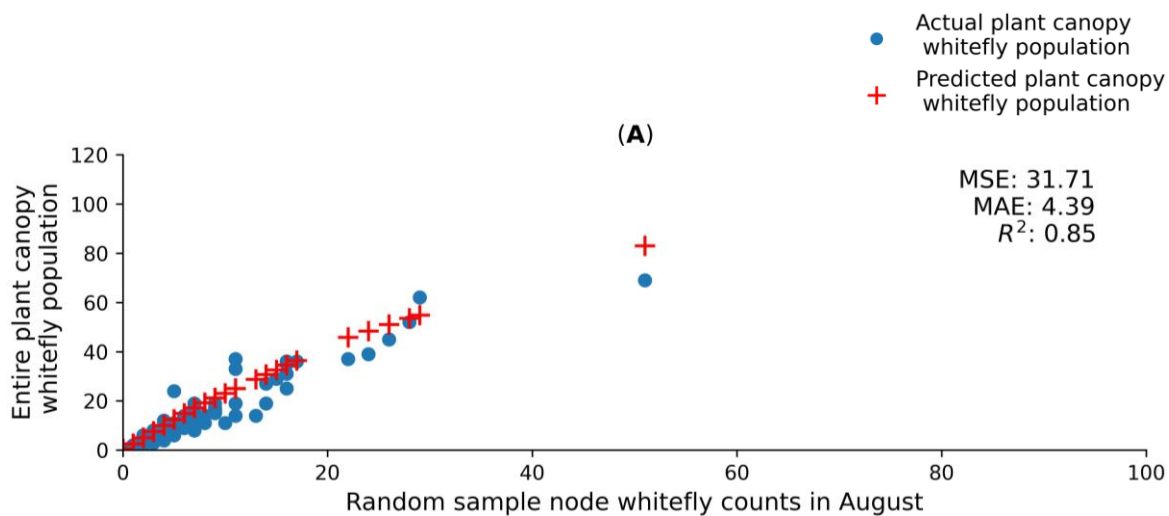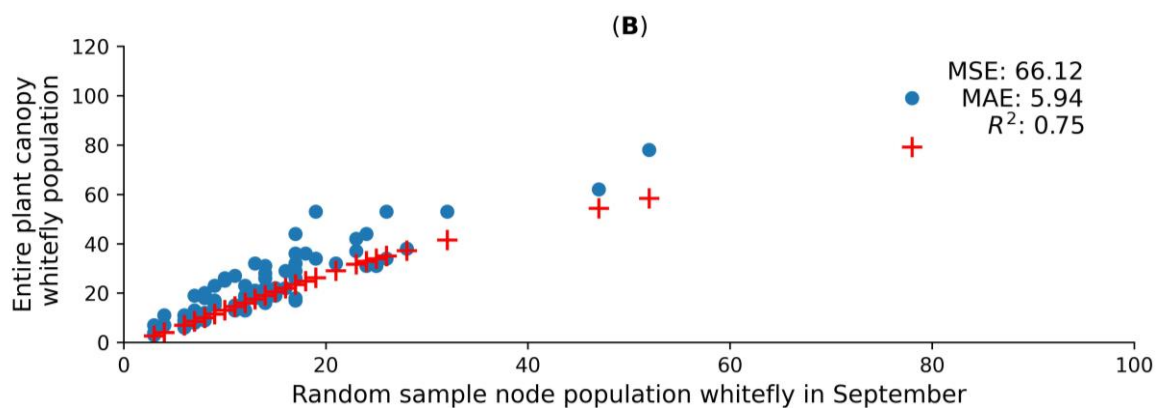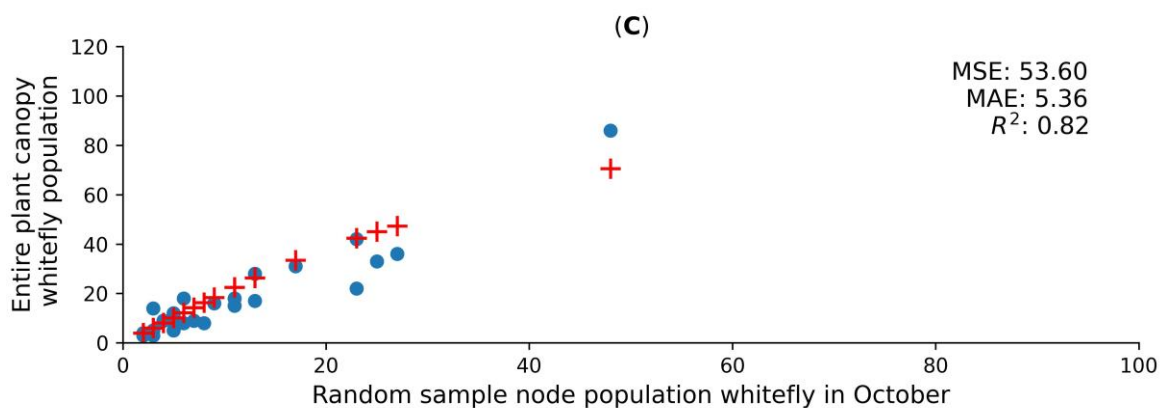

Supplement: toaf035_suppl_Supplementary_Material [file toaf035_suppl_supplementary_material.zip › Supplementary Fig S4.pdf]

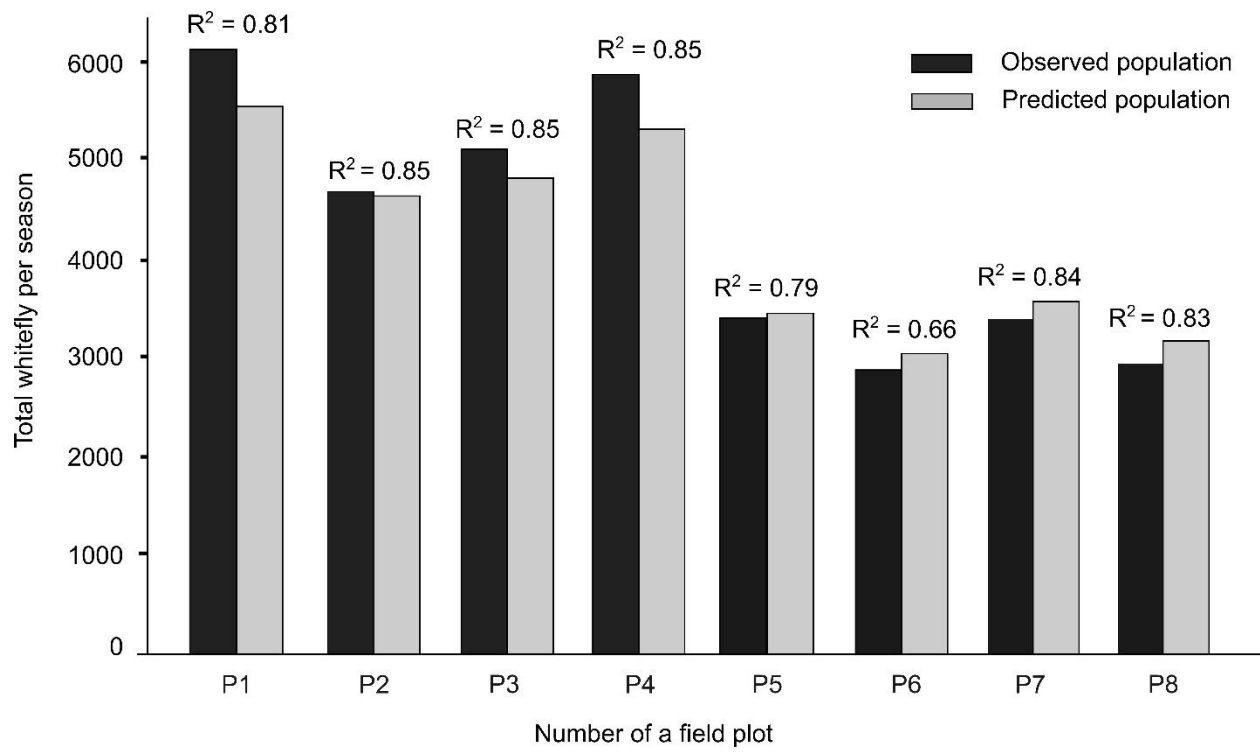

Supplement: toaf035_suppl_Supplementary_Material [file toaf035_suppl_supplementary_material.zip › Supplementary Fig S5.pdf]
